# Gastric bacterial microbiota and gastric carcinogenesis: from dysbiosis to immune remodeling and therapeutic opportunities

**DOI:** 10.3389/fimmu.2026.1953049

**Published:** 2026-09-14

**Authors:** Shengyu Huang, Yiwen Cheng, Yangtian Chen, Yang Liu, Xia Liu, Xiaocui Xu, Pingping Hu, Lingbin Wu, Zongxin Ling, Lulu Xu

**Affiliations:** 1 State Key Laboratory for Diagnosis and Treatment of Infectious Diseases, National Clinical Research Center for Infectious Diseases, National Medical Center for Infectious Diseases, Collaborative Innovation Center for Diagnosis and Treatment of Infectious Diseases, The First Affiliated Hospital, Zhejiang University School of Medicine, Hangzhou, Zhejiang, China; 2 Department of Anesthesiology, Affiliated Hospital of Nantong University, Medical School of Nantong University, Nantong, China; 3 Department of Laboratory Medicine, Huludao Central Hospital, Huludao, Liaoning, China; 4 Department of Intensive Care Unit, The First Affiliated Hospital, Zhejiang University School of Medicine, Hangzhou, Zhejiang, China; 5 Department of Laboratory Medicine, Lishui Second People’s Hospital, Lishui, Zhejiang, China

**Keywords:** dysbiosis, gastric carcinogenesis, gastric microbiota, *Helicobacter pylori*, immune remodeling, immunotherapy, probiotics, tumor microenvironment

## Abstract

Gastric cancer remains a leading cause of cancer-related mortality worldwide. The classical *Helicobacter pylori*-centric paradigm of gastric carcinogenesis is being progressively expanded, as it is now recognized that the stomach harbors a complex and metabolically active microbial ecosystem whose compositional shifts fundamentally influence oncogenic progression. This review proposes a unifying dysbiosis-carcinogenesis-immune remodeling-therapeutic intervention (DCIT) framework to integrate the rapidly growing body of evidence linking gastric microbiota dysbiosis to malignancy. We survey the compositional landscape of the gastric microbiota across the Correa cascade, from healthy homeostasis through atrophic gastritis, intestinal metaplasia, and adenocarcinoma, and examine the ecological succession that releases niche constraints for oral commensals and nitrate-reducing taxa in advanced disease stages. We systematically evaluate four complementary mechanistic axes of microbial carcinogenesis: genotoxicity driven by bacterial effectors, chronic inflammation sustained by pattern recognition receptor signaling, epithelial barrier disruption facilitating bacterial translocation, and metabolic reprogramming of the gastric niche generating carcinogenic *N*-nitroso compounds and secondary bile acids. The review further examines how gastric microbiota remodels the tumor immune microenvironment through immune cell reprogramming, metabolite-mediated signaling, and checkpoint modulation, with direct implications for the efficacy of immune checkpoint inhibitor immunotherapy. The translational pipeline is evaluated from established *H. pylori* eradication regimens through probiotics, phage therapy, and fecal microbiota transplantation to emerging strategies that integrate microbiota modulation with chemotherapy and immunotherapy. Finally, we assess the methodological challenges of low-biomass gastric microbiome research, the integration of multi-omics platforms, and the critical evidence gaps that need to be addressed to realize the clinical potential of microbiota-targeted strategies for gastric cancer prevention and treatment.

## 1 Introduction

Gastric cancer (GC) ranks as the fifth most common malignancy and the fourth leading cause of cancer-related mortality worldwide, accounting for approximately 770,000 deaths annually (1–3). The classical etiological framework has centered primarily on Helicobacter pylori, a chronic gastric colonizer designated a Group I carcinogen by the International Agency for Research on Cancer, whose sustained infection drives the stepwise histopathological progression, the Correa cascade, from chronic superficial gastritis through atrophic gastritis, intestinal metaplasia, and dysplasia to invasive adenocarcinoma (4–6). Two observations however, have challenged the adequacy of this *H. pylori*-centric model. First, only 1-3% of *H. pylori*-infected individuals ever develop GC, indicating that additional microbial, host, and environmental factors modulate carcinogenic risk (7–9). Second, although *H. pylori* eradication reduces GC incidence by approximately 35-50%, it does not eliminate the risk entirely, especially in patients who have already developed advanced preneoplastic lesions (10, 11).

The advent of culture-independent sequencing technologies has overturned the long-held dogma that the human stomach is sterile. High-throughput 16S ribosomal RNA (rRNA) g1ene sequencing and shotgun metagenomics have revealed that the gastric mucosa harbors a phylogenetically diverse microbial community whose composition is profoundly altered along the Correa cascade (4, 12, 13). This remodeling unfolds as a progressive loss of microbial diversity and enrichment of oral-origin taxa in advanced disease stages (14–16). Such compositional shifts are increasingly recognized not merely as epiphenomena of the changing gastric environment but as active contributors to carcinogenesis through genotoxic, inflammatory, and immunomodulatory mechanisms (17–19).

This review primarily focuses on the bacterial component of the gastric microbiota, for which the evidence linking dysbiosis to gastric carcinogenesis is most abundant and which remains the most extensively characterized fraction of the gastric microbiome; the gastric mycobiome and virome are addressed where relevant, although the mechanistic framework developed here is built primarily on bacterial communities. To organize this rapidly expanding evidence base, we propose a unifying framework, termed dysbiosis-carcinogenesis-immune remodeling-therapeutic intervention (DCIT), comprising four interconnected analytical dimensions, each evaluated against the classical *H. pylori*-centric paradigm: the ecological dynamics of the gastric microbiota along the health-to-cancer continuum and the ecological drivers of community restructuring; the molecular mechanisms of microbial-driven carcinogenesis across four complementary axes, including genotoxicity, chronic inflammation, epithelial barrier disruption, and metabolic reprogramming; the remodeling of the tumor immune microenvironment (TIME) by gastric microbiota, which shapes the balance between antitumor immunity and immune evasion; and the translational pipeline of microbiota-targeted interventions, from established *H. pylori* eradication strategies to emerging modalities including phage therapy, engineered probiotics, and microbiota-informed immunotherapy. Across all dimensions, we emphasize the evidence hierarchy distinguishing *H. pylori*-dependent from *H. pylori*-independent pathways, the methodological challenges inherent to low-biomass gastric microbiome research, and the translational gaps that remain to be overcome before microbiota-based diagnostics and therapeutics can enter clinical practice (20–23).

Taken together, these four dimensions constitute a conceptual scaffold for understanding how gastric microbial dysbiosis initiates and sustains the multistage carcinogenic process (**Figure 1**). The framework posits that dysbiosis is not merely a consequence of the changing gastric microenvironment but a driver that amplifies each subsequent stage: microbial community restructuring generates genotoxic and inflammatory stimuli (carcinogenesis), which in turn reprogram the immune landscape (immune remodeling) and create a permissive niche for tumor progression (24–26). Each dimension thereby reveals nodes at which therapeutic intervention can disrupt the carcinogenic cascade, from upstream microbiota restoration to downstream immune checkpoint modulation. The following sections evaluate the evidence for each DCIT dimension in sequence, moving from the compositional characterization of the gastric microbiota through the mechanistic dissection of carcinogenic pathways and immune remodeling to therapeutic translation.

**Figure 1:**
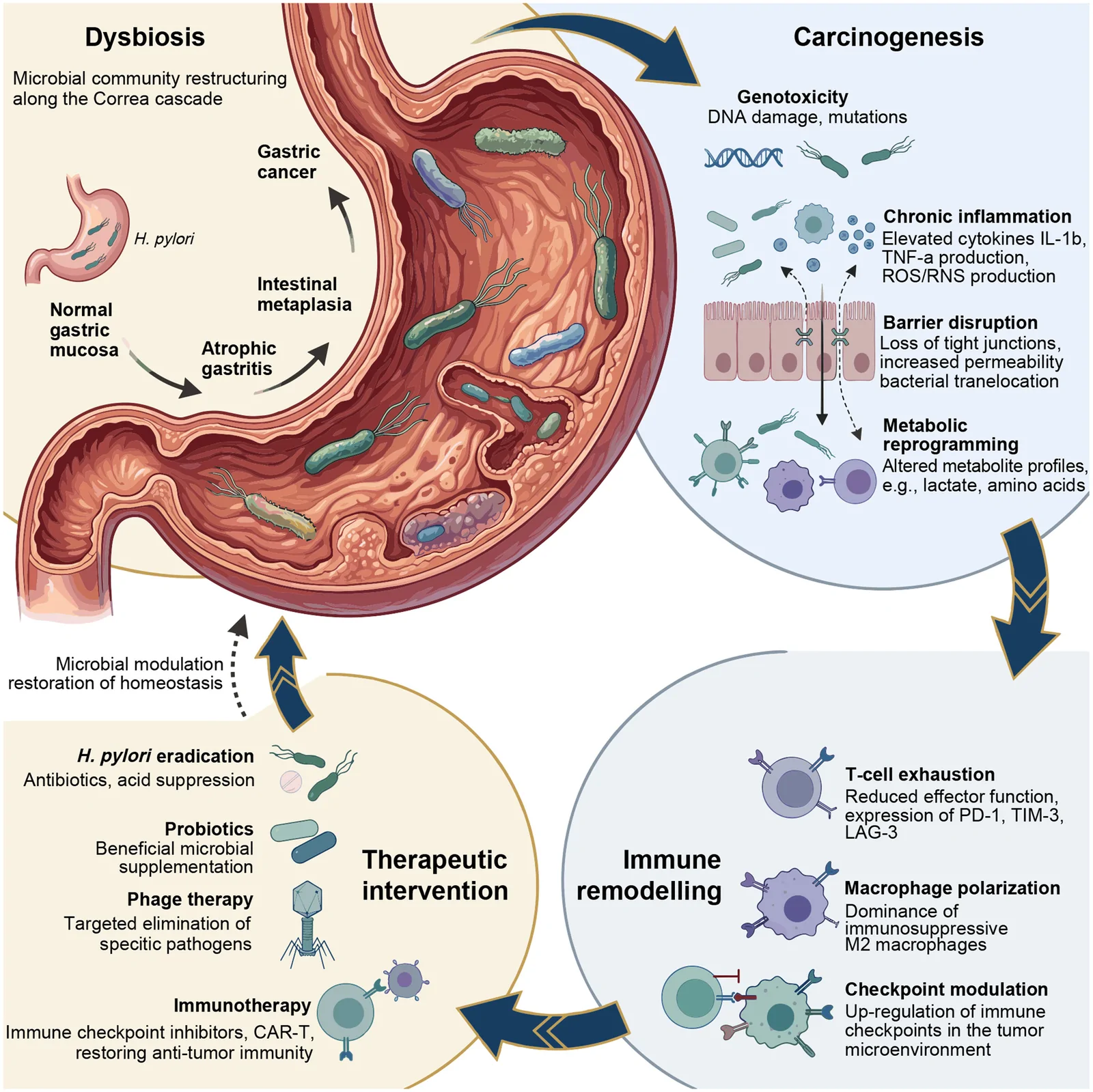
The DCIT framework. The diagram integrates gastric microbiota dysbiosis, carcinogenesis mechanisms, immune remodeling, and therapeutic interventions. The central cascade depicts microbial succession along the Correa cascade, from *H. pylori* dominance to enrichment of oral commensals and nitrate-reducing taxa in advanced lesions. Arrows indicate feed-forward loops among dysbiosis, inflammation, immune suppression, and niche modification. The outer ring highlights potential intervention points, including *H. pylori* eradication, probiotics, phage therapy, FMT, engineered probiotics, and immune checkpoint blockade.

## 2 The gastric microbiota landscape: from homeostasis to dysbiosis

The gastric microbiota is a low-biomass yet phylogenetically diverse microbial ecosystem whose composition is governed by the extreme physicochemical conditions of the stomach, including high acidity, dynamic mucus layer architecture, and efficient peristaltic clearance (4, 27, 28). The stomach’s harsh environment imposes strong selective pressure on microbial colonization, yet a distinct core community has adapted to this niche. Understanding the compositional dynamics of this ecosystem across the spectrum of gastric health and disease is essential to the DCIT framework, as dysbiotic community restructuring represents the first dimension from which carcinogenic and immunomodulatory consequences arise (29, 30).

### 2.1 Compositional profiling of the healthy gastric microbiome

Gastric acid secretion, mediated by parietal cell H^+^/K^+^-ATPase activity, maintains a luminal pH that is bactericidal for most ingested microorganisms (28, 31). The gastric mucus layer, composed of heavily glycosylated MUC5AC and MUC6 mucins, provides a pH gradient from the acidic lumen (pH 1-2) to the near-neutral epithelial surface (pH 6-7), creating a sheltered niche for acid-sensitive microbes (32, 33). The mucus layer also provides nutrients for mucin-degrading bacteria and serves as an adhesion substrate for microbial colonization. In healthy individuals, the gastric microbiota is thought to be transiently replenished by swallowed oral and nasopharyngeal microbes, with approximately 103–104 colony-forming units per milliliter of gastric fluid (34–36). The balance among microbial influx, gastric clearance, and niche-specific colonization determines the steady-state composition of the gastric microbial community.

At the phylum level, the healthy gastric microbiota is dominated by Proteobacteria, Firmicutes, Bacteroidetes, and Actinobacteria, with Fusobacteria and candidate phyla detected at lower abundance (4, 12, 34). The most consistently detected genera include Prevotella, Streptococcus, Veillonella, Neisseria, Haemophilus, and Lactobacillus (37–39). Despite considerable inter-individual variability, the core gastric microbiota in *H. pylori*-negative healthy individuals appears to be enriched in species capable of surviving acidic conditions, including Lactobacillus and Streptococcus species, both of which produce lactic acid and may employ additional acid-tolerance mechanisms such as proton pumps to maintain intracellular pH homeostasis (40, 41). This variability reflects differences in gastric acid output, dietary habits, age, proton pump inhibitor (PPI) use, and geographic origin (42–44). A systematic review and meta-analysis of gastric mucosal microbiota studies further reported substantial interstudy heterogeneity in the composition of the healthy gastric microbiome, attributable partly to differences in sampling site (antrum versus corpus), biopsy processing, and sequencing platform (38). Despite these methodological challenges, the distinctive ecological pressures of the gastric environment select for a resilient, acid-tolerant microbial assemblage that establishes the baseline from which dysbiotic shifts later emerge.

### 2.2 *H. pylori*-driven microbial restructuring

*H. pylori* colonization fundamentally restructures the gastric microbial ecosystem. As a urease-producing bacterium, *H. pylori* hydrolyzes urea to ammonia and carbon dioxide, thereby neutralizing the gastric acid in its immediate microenvironment and creating a less acidic niche permissive for secondary microbial colonizers (31, 45, 46). This pH-modulating effect is the primary mechanism by which *H. pylori* acts as a keystone pathobiont, altering the ecological conditions that govern gastric microbial community assembly. In colonized individuals, the relative abundance of Proteobacteria rises dramatically, driven predominantly by *H. pylori* itself, which can account for 40-90% of the gastric microbial community in colonized individuals (4, 13). This dominance is accompanied by reduced alpha diversity and altered relative abundances of Firmicutes, Bacteroidetes, and Actinobacteria (14, 15, 47).

The magnitude of *H. pylori*-driven microbial restructuring correlates with the virulence factor repertoire of the infecting strain. Strains harboring the cag pathogenicity island (cagPAI) are associated with more pronounced gastric inflammation and more extensive disruption of the gastric microbiota (48, 49). The vacuolating cytotoxin VacA further alters the gastric epithelial niche and may differentially affect the growth of competing bacterial species (50–52). *H. pylori* also competes with other gastric microbes for adhesion sites and essential nutrients including iron, thereby shaping the composition of the co-colonizing microbial community (53, 54).

In addition to reshaping the microbial community through pH modulation and competition, *H. pylori* virulence factors also trigger a chronic inflammatory response that alters the gastric microenvironment in ways favoring specific bacterial taxa. The *H. pylori*-driven infiltration of neutrophils, lymphocytes, and macrophages into the gastric mucosa is accompanied by increased production of reactive oxygen and nitrogen species, which can serve as terminal electron acceptors for nitrate- and nitrite-reducing bacteria (55, 56). This creates a selective advantage for taxa such as Veillonella, Neisseria, and Haemophilus, which are enriched in the *H. pylori*-positive stomach and may contribute to the generation of carcinogenic N-nitroso compounds (18, 57, 58).

### 2.3 Dysbiotic trajectories along the Correa cascade

The progression of gastric disease along the Correa cascade is accompanied by a characteristic pattern of microbial community restructuring, which has been documented across multiple independent cohorts (14, 38, 59). This trajectory is not linear; rather, it reflects an ecological succession driven by the progressive loss of gastric acid secretion, remodeling of the gastric mucus layer, and changes in metabolic substrate availability as the gastric epithelium undergoes metaplastic transformation (60–62).

In the early stages of the Correa cascade (chronic superficial gastritis and early atrophic gastritis), *H. pylori* remains the dominant microbial constituent, and the gastric microbiota is characterized by low alpha diversity and high *H. pylori* relative abundance (4, 13). As parietal cell loss progresses and gastric acid secretion declines, the stomach becomes increasingly permissive for microbial colonization. This transition is marked by a paradoxical increase in alpha diversity and a shift in community composition away from *H. pylori* dominance toward a more diverse community enriched in oral-origin taxa (14, 15). In advanced atrophic gastritis and intestinal metaplasia, the gastric environment becomes hypochlorhydric or achlorhydric, and the microbiota increasingly resembles that of the oral cavity and upper small intestine, with enrichment of Fusobacterium, Peptostreptococcus, Prevotella, and Veillonella species (63–66).

The ecological succession model proposes that *H. pylori* depletion during advanced preneoplastic stages releases niche constraints that previously limited the growth of competing bacterial taxa (17, 18). *H. pylori* itself may become undetectable in the atrophic stomach, as the loss of gastric epithelial cells and the altered mucosal environment render the gastric niche inhospitable to continued *H. pylori* colonization. This creates an ecological vacuum that is filled by oral commensals, nitrate-reducing bacteria, and lactic acid-producing taxa, many of which possess metabolic capabilities that can contribute to carcinogenesis independently of *H. pylori* (7, 67, 68). The resulting dysbiotic community is characterized by increased abundance of Fusobacterium nucleatum, a Gram-negative oral anaerobe associated with poor prognosis in multiple gastrointestinal cancers (69, 70), and by enrichment of nitrate-reducing taxa including Veillonella, Neisseria, and Haemophilus species which can generate carcinogenic N-nitroso compounds from dietary nitrates (18, 57).

The gastric microbiota composition in gastric adenocarcinoma is distinct from that in non-malignant gastric tissue, with consistent differences reported in both the mucosal and luminal compartments (71, 72). A meta-analysis of gastric mucosal microbiota studies identified several genera that were differentially abundant in GC compared with non-cancer controls, including enrichment of Fusobacterium, Lactobacillus, Streptococcus, and Veillonella and depletion of Helicobacter and Prevotella (14). Shotgun metagenomic analysis of gastric adenocarcinoma tissue has further revealed functional shifts in the gastric microbiome, including enrichment of pathways involved in nitrate reduction, lipopolysaccharide biosynthesis, and flagellar motility, consistent with a procarcinogenic functional profile (73–75). These findings support the hypothesis that the gastric microbiota in GC is not merely a passive reflection of the TIME but an active participant in disease progression (76–78).

Methodological considerations are critical for interpreting gastric microbiota studies. The low-biomass nature of gastric samples renders them susceptible to contamination from reagents, laboratory consumables, and the oral cavity during endoscopic sampling (4, 17). Negative controls, including sterile biopsy forceps rinses and DNA extraction blank controls, are essential to distinguish genuine gastric microbial signals from contaminating sequences. The choice of sequencing platform (16S rRNA gene sequencing versus shotgun metagenomics) also influences the resolution and functional information obtained. 16S rRNA gene sequencing provides taxonomic profiling at the genus level and is cost-effective for large cohort studies, whereas shotgun metagenomics enables species-level resolution and functional gene annotation but is more expensive and computationally demanding (17, 75, 79). Complementary application of both approaches, combined with rigorous contamination controls, is recommended to maximize the reliability and interpretability of gastric microbiome data.

The progressive dysbiosis observed along the Correa cascade (summarized in **Table 1**) reveals a pattern of ecological succession in which the depletion of *H. pylori* during advanced stages releases niche constraints and enables the overgrowth of oral commensals and nitrate-reducing taxa (14, 18). This compositional shift is not merely a marker of disease progression but carries functional consequences that directly contribute to carcinogenesis (18, 57, 70, 80, 81). The transition from a low-diversity, *H. pylori*-dominated community to a high-diversity, oral-commensal-dominated community thus represents a critical inflection point in the DCIT framework, linking the first dimension of dysbiosis to the second dimension of carcinogenesis.

**Table 1:** Gastric microbiota composition across stages of the Correa cascade.

Disease stage	Dominant phyla	Key genera enriched	α-diversity	Metabolic features
Healthy (Hp–)	Proteobacteria, Firmicutes	*Prevotella*, *Streptococcus*	Moderate	Acid-tolerant taxa
Chronic gastritis (Hp+)	Proteobacteria	*Helicobacter*	Low	Urease activity dominant
Atrophic gastritis	Firmicutes, Bacteroidetes	*Streptococcus*, *Veillonella*	Increasing	Nitrate reduction
Intestinal metaplasia	Firmicutes, Fusobacteria	*Fusobacterium*, *Peptostreptococcus*	High	Mucin degradation
Adenocarcinoma	Firmicutes, Fusobacteria	*Fusobacterium*, *Lactobacillus*	High	*N*-nitroso compound generation

## 3 Mechanisms of microbial-driven gastric carcinogenesis

The compositional shifts described in the preceding section are not epiphenomena; rather, they represent the first dimension of the DCIT framework from which the second dimension, carcinogenesis, directly emerges. Microbial-driven gastric carcinogenesis proceeds through four complementary mechanistic axes that are progressively activated along the Correa cascade: genotoxicity mediated by bacterial effectors, chronic inflammation sustained by innate immune signaling, epithelial barrier disruption facilitating bacterial translocation, and metabolic reprogramming of the gastric niche (7, 55, 82). These axes do not operate in isolation; they intersect and amplify one another, creating a feed-forward carcinogenic loop that progressively drives gastric epithelial cells toward malignant transformation (25, 78, 83).

### 3.1 Genotoxicity and epigenetic remodeling by bacterial effectors

The most direct mechanism by which gastric microbes promote carcinogenesis is the induction of DNA damage. *H. pylori* CagA, the best-characterized bacterial oncoprotein, is translocated into gastric epithelial cells via the cagPAI-encoded type IV secretion system, where it undergoes tyrosine phosphorylation and interacts with multiple host signaling proteins, including SHP-2 phosphatase, Grb2, and c-Met (84, 85). CagA-mediated activation of SHP-2 leads to sustained Erk/MAPK signaling, which promotes epithelial proliferation and simultaneously induces replication stress and DNA double-strand breaks through the generation of reactive oxygen species (ROS) (55, 86, 87). CagA also disrupts the apical junctional complex and activates β-catenin signaling, further contributing to genomic instability via accumulation of β-catenin-driven proliferative signals and the suppression of DNA damage repair pathways (5, 82, 88).

The vacuolating cytotoxin VacA complements CagA-mediated genotoxicity through distinct mechanisms. VacA forms anion-selective channels in the mitochondrial membrane, leading to mitochondrial depolarization, cytochrome c release, and generation of mitochondrial ROS (50, 51, 89). VacA-induced mitochondrial dysfunction not only triggers apoptosis in a subset of gastric epithelial cells but also generates sublethal DNA damage in surviving cells, contributing to the accumulation of mutations over time (52, 55). VacA also potentiates CagA-mediated signaling: it promotes CagA accumulation in gastric epithelial cells by inhibiting autophagic degradation of the oncoprotein, thereby amplifying CagA-mediated genotoxic signaling (50, 53, 54).

Beyond *H. pylori*, other gastric bacteria harbor genotoxic capabilities. Escherichia coli strains carrying the pks genomic island produce colibactin, a genotoxin that induces DNA interstrand crosslinks and double-strand breaks in eukaryotic cells (7, 90, 91). Although colibactin-producing E. coli are primarily associated with colorectal cancer, they have been detected in the gastric mucosa of GC patients, suggesting a potential contribution to gastric carcinogenesis. Cytolethal distending toxin (CDT), produced by several Gram-negative bacteria including Campylobacter and certain E. coli strains, induces DNA double-strand breaks via its DNase activity after translocation into the host cell nucleus (25, 78). F. nucleatum, enriched in the GC microbiota, produces FadA adhesin, which binds to E-cadherin on epithelial cells and activates β-catenin signaling, promoting epithelial proliferation and potentially contributing to genomic instability through replication stress (69, 70, 92, 93).

Epigenetic remodeling constitutes an additional layer of microbial-driven genomic alteration. *H. pylori* infection induces aberrant DNA methylation in gastric epithelial cells, including hypermethylation of CpG islands in the promoters of tumor suppressor genes such as CDH1 (encoding E-cadherin), MLH1, and CDKN2A (55, 94, 95). This epigenetic silencing is mediated in part by *H. pylori*-induced upregulation of the DNA methyltransferases DNMT1 and DNMT3B through NF-κB-dependent signaling (82, 96). Importantly, *H. pylori*-associated methylation patterns can persist after eradication, creating a “field defect” of epigenetic alterations that may explain the continued risk of GC despite successful bacterial clearance (10, 97, 98). Epstein-Barr virus (EBV), which is associated with approximately 10% of GC cases, drives a distinct pattern of extensive CpG island methylation (the CpG island methylator phenotype, or CIMP), thereby silencing multiple tumor suppressor genes and contributing to the molecular heterogeneity of GC (99, 100).

### 3.2 Chronic inflammation and innate immune signaling

The second mechanistic axis of microbial carcinogenesis is sustained chronic inflammation, initiated when microbial ligands are recognized by pattern recognition receptors (PRRs) on gastric epithelial cells and innate immune cells. Toll-like receptors (TLRs) and NOD-like receptors (NLRs) are the primary PRR families involved in sensing gastric microbial products. TLR2, TLR4, and TLR5 recognize bacterial lipoproteins, lipopolysaccharide (LPS), and flagellin, respectively, while NOD1 and NOD2 detect peptidoglycan fragments from Gram-negative and Gram-positive bacteria (101–103).

*H. pylori* LPS, although less endotoxic than that of Enterobacteriaceae, engages TLR4 on gastric epithelial cells and macrophages, thereby activating NF-κB and MAPK signaling cascades that drive the transcription of pro-inflammatory cytokines including IL-1β, IL-6, IL-8, and TNF-α (56, 82). NOD1 recognition of *H. pylori* peptidoglycan delivered via the cagPAI type IV secretion system represents a second major innate immune activation pathway, contributing to recruitment of neutrophils and establishment of chronic active gastritis (101, 104). The resulting inflammatory milieu is characterized by sustained NF-κB activation, which promotes epithelial proliferation, suppresses apoptosis through the induction of anti-apoptotic proteins such as Bcl-2 and Bcl-xL, and generates a microenvironment rich in ROS and reactive nitrogen species that cause cumulative DNA damage (55, 96, 105).

The IL-1β/IL-6/IL-17 cytokine axis is central to the pro-carcinogenic inflammatory response in the stomach. IL-1β, produced by activated macrophages and dendritic cells in response to *H. pylori* and other microbial stimuli, is a potent pro-inflammatory cytokine that promotes Th17 cell differentiation and inhibits gastric acid secretion, thereby creating a feedback loop that further alters the gastric microbial niche (55, 82, 106). Genetic polymorphisms in the IL-1β gene cluster (IL-1B encoding IL-1β and IL-1RN encoding IL-1 receptor antagonist) that result in higher IL-1β production are associated with increased GC risk, particularly in *H. pylori*-infected individuals (7, 84). IL-6, produced by gastric epithelial cells and infiltrating immune cells in response to TLR and NF-κB signaling, activates STAT3, which promotes epithelial proliferation, survival, and angiogenesis (61, 107). IL-17, secreted by Th17 cells and γδT cells in the *H. pylori*-infected gastric mucosa, recruits neutrophils and amplifies the inflammatory response, contributing to the sustained tissue damage that drives the Correa cascade (103, 108).

Non-*H. pylori* gastric microbes contribute to the inflammatory milieu through distinct PRR activation pathways. F. nucleatum LPS engages TLR4 with higher potency than *H. pylori* LPS in some experimental systems, potentially amplifying the inflammatory response in the GC microenvironment (104, 109). The enrichment of Gram-negative taxa in the dysbiotic gastric microbiota increases the total load of LPS and other TLR ligands in the gastric mucosa, sustaining NF-κB activation even in the absence of *H. pylori*. The cGAS-STING pathway, which senses cytosolic double-stranded DNA, constitutes an additional innate immune axis that may be activated by bacterial DNA released during infection or by mitochondrial DNA released from damaged epithelial cells (105, 110). STING activation in gastric epithelial cells and infiltrating immune cells can have dual consequences: acute STING signaling promotes type I interferon responses and antitumor immunity, while chronic STING activation has been associated with immunosuppression and tumor promotion in some contexts (101, 111).

### 3.3 Epithelial barrier disruption and bacterial translocation

The integrity of the gastric epithelial barrier is a critical determinant of the spatial relationship between the gastric microbiota and the host immune system. *H. pylori* CagA directly disrupts epithelial barrier function by targeting the tight junction proteins ZO-1 and occludin, thereby increasing paracellular permeability (55, 87). CagA-mediated disruption of the E-cadherin/β-catenin complex at adherens junctions further compromises epithelial integrity, while VacA-induced epithelial cell apoptosis creates focal disruptions in the epithelial monolayer (50, 89). The combined effect of these virulence factor activities is a gastric epithelium with increased permeability to luminal contents, including microbial products and intact bacteria (82, 112).

Dysbiosis-associated degradation of the gastric mucus layer amplifies barrier disruption. The gastric mucus layer is a dynamic structure whose thickness and composition are modulated by microbial activity. Mucin-degrading bacteria, including certain Bacteroides and Akkermansia species, express mucinases that cleave the glycosylated domains of MUC5AC and MUC6, thereby thinning the protective mucus gel and exposing the underlying epithelium to direct microbial contact (32, 33, 113). The loss of mucus barrier integrity is particularly relevant in the context of atrophic gastritis, where replacement of gastric epithelium by intestinal-type epithelium (intestinal metaplasia) alters the mucin expression profile and may further compromise barrier function.

Increased epithelial permeability facilitates bacterial translocation into the lamina propria, where microbial products engage TLRs and NLRs on submucosal macrophages, dendritic cells, and stromal cells, triggering a submucosal inflammatory response that is spatially and qualitatively distinct from the superficial epithelial inflammation observed in early *H. pylori* gastritis (66, 76). Bacterial translocation also exposes the adaptive immune system to gastric microbial antigens, potentially shaping the tumor-directed immune response through mechanisms that intersect with the immune remodeling dimension of the DCIT framework (114–116). Translocation of oral commensals, including F. nucleatum, into the gastric submucosa may be particularly immunogenic, given the potent TLR4 and TLR2 agonist activity of these organisms (117).

### 3.4 Microbial metabolic reprogramming of the gastric niche

The fourth mechanistic axis of microbial carcinogenesis is metabolic reprogramming: transformation of the gastric metabolic environment by microbial enzyme activities that generate carcinogenic compounds. This axis is particularly relevant in the context of advanced gastric atrophy and intestinal metaplasia, where a hypochlorhydric environment combined with enrichment of nitrate-reducing bacteria creates conditions favorable for generation of N-nitroso compounds (18, 66).

Dietary nitrates, abundant in vegetables and preserved meats, are absorbed in the small intestine and concentrated in saliva, where oral nitrate-reducing bacteria convert them to nitrite. In the healthy, acidic stomach, salivary nitrite is rapidly protonated to nitrous acid and decomposed to nitric oxide and other reactive nitrogen species, which have antimicrobial activity. In the hypochlorhydric stomach, however, nitrite is not efficiently decomposed and instead accumulates, providing a substrate for gastric nitrate-reducing and nitrite-reducing bacteria (18, 66). These bacteria, including Veillonella, Neisseria, Haemophilus, and Staphylococcus species, catalyze reduction of nitrite to nitric oxide and formation of N-nitroso compounds through reaction of nitrite-derived nitrosating agents with secondary amines and amides present in the gastric lumen (83, 118). N-nitroso compounds are potent alkylating agents that induce DNA adducts and mutations, and their sustained generation in the hypochlorhydric stomach has been proposed as a key mechanism of non-*H. pylori*-driven gastric carcinogenesis (119, 120).

Short-chain fatty acids (SCFAs), including acetate, propionate, and butyrate, are produced by bacterial fermentation of dietary fiber and are present in the gastric lumen at concentrations that vary with microbial composition and diet. In the colonic epithelium, butyrate serves as the primary energy source for colonocytes and has well-characterized anti-inflammatory and tumor-suppressive effects. In the gastric epithelium, however, the role of SCFAs is more complex. Butyrate has been shown to inhibit *H. pylori*-induced NF-κB activation in gastric epithelial cells and to reduce expression of pro-inflammatory cytokines, suggesting a protective role (121, 122). Conversely, in the context of established dysplasia or carcinoma, butyrate may promote tumor progression through its effects on histone deacetylase inhibition and cell cycle gene regulation, highlighting the context-dependent nature of SCFA effects in gastric carcinogenesis (121, 123, 124).

Bile acid dysmetabolism represents an additional metabolic axis. In the healthy stomach, bile acids are present at low concentrations and are primarily in conjugated form. In the presence of gastric dysbiosis, particularly in patients with duodenogastric reflux, bile acids are deconjugated and dehydroxylated by bacterial enzymes, generating secondary bile acids including deoxycholic acid and lithocholic acid (125, 126). Secondary bile acids are genotoxic and pro-inflammatory, inducing DNA damage, mitochondrial dysfunction, and NF-κB activation in gastric epithelial cells (118, 127). Enrichment of bile acid-metabolizing bacteria in the gastric microbiota of GC patients has been reported, suggesting a mechanistic link between gastric dysbiosis and carcinogenesis (128, 129).

The four mechanistic axes are not independent; they form an interconnected carcinogenic network (**Figure 2**). Genotoxic DNA damage triggers inflammatory signaling through the activation of DNA damage response pathways that converge on NF-κB (55, 82). Chronic inflammation, in turn, generates ROS that amplify DNA damage and promotes epithelial proliferation, expanding the pool of cells at risk for malignant transformation (96, 101). Epithelial barrier disruption allows microbial products to access the submucosal compartment, amplifying the inflammatory response. Metabolic reprogramming generates genotoxic compounds that sustain DNA damage independently of the inflammatory axis. This network architecture implies that targeting any single axis may be insufficient to interrupt the carcinogenic process, and therapeutic strategies addressing multiple axes simultaneously are likely to be most effective (129).

**Figure 2:**
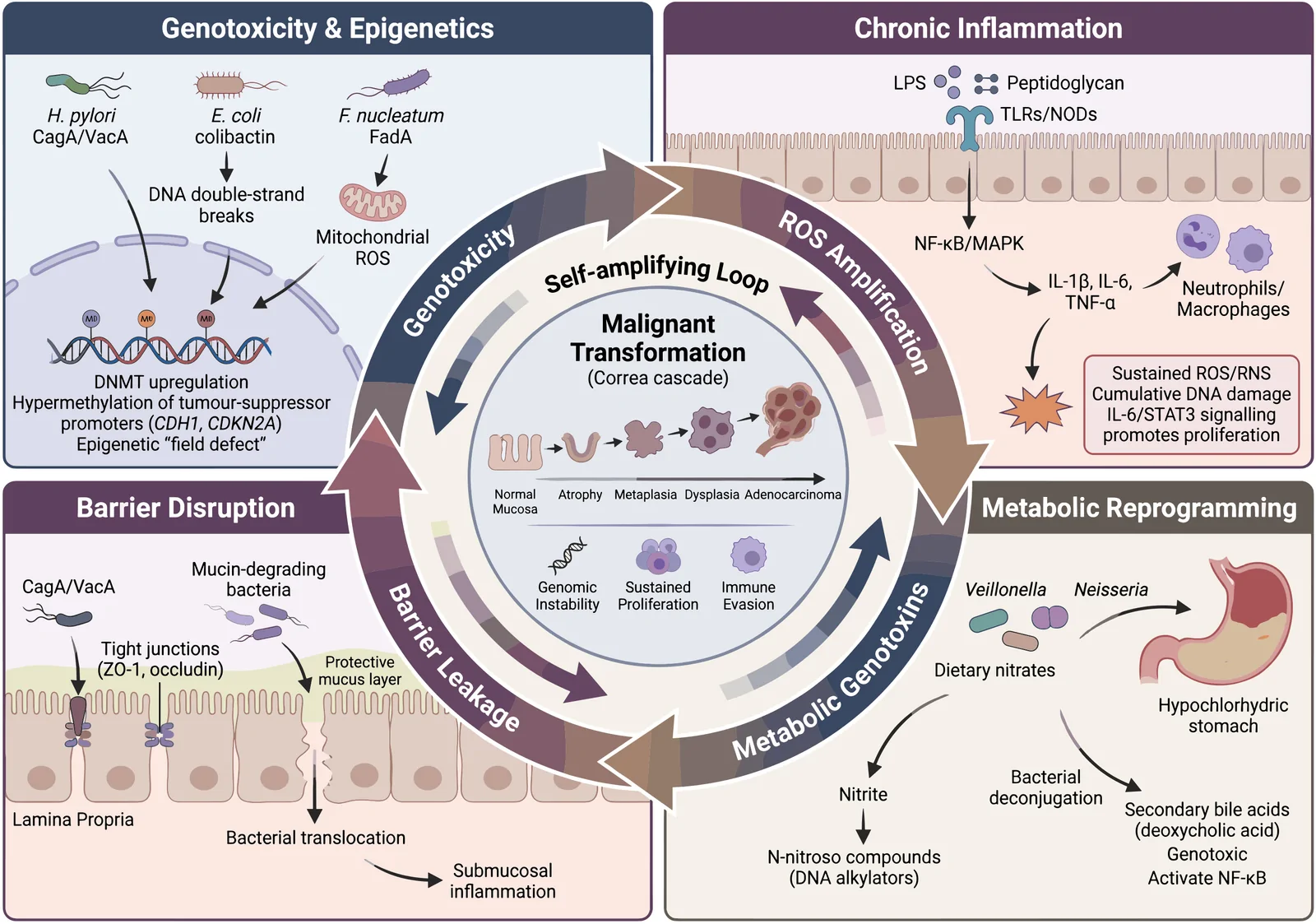
Four mechanistic axes of microbial-driven gastric carcinogenesis. The schematic shows genotoxicity (bacterial effectors such as CagA, VacA, colibactin, and CDT), chronic inflammation (TLR/NLR signaling and NF-κB activation), epithelial barrier disruption (tight junction and mucus layer degradation), and metabolic reprogramming (generation of N-nitroso compounds and secondary bile acids). These axes are interconnected and reinforce one another, forming a self-amplifying pro-carcinogenic network.

## 4 Immune remodeling of the gastric TIME

Having established the four mechanistic axes through which gastric microbes drive carcinogenesis, the third dimension of the DCIT framework now addresses how gastric microbial communities reshape the TIME—a process that critically determines the balance between antitumor immunosurveillance and immune evasion (114, 116, 130). The gastric TIME is a complex ecosystem comprising tumor cells, stromal cells, blood and lymphatic vessels, and a diverse array of immune cells whose composition and functional polarization are influenced by the gastric microbiota (131–134). Understanding how microbial communities modulate the TIME is crucial for predicting the efficacy of immunotherapeutic interventions and for designing microbiota-informed strategies to enhance antitumor immunity (135, 136).

### 4.1 Immune cell landscape reprogramming by gastric microbiota

In GC, the gastric microbiota shapes the composition and functional polarization of tumor-infiltrating immune cells via multiple mechanisms: direct bacterial interaction with immune cells, microbial metabolite-mediated signaling, and modulation of chemokine and cytokine gradients within the TIME (137). Single-cell RNA sequencing studies of gastric tumors have revealed a complex immune cell landscape comprising CD8+ cytotoxic T cells, CD4+ helper T cells (including Th1, Th2, Th17, and Treg subsets), B cells, natural killer (NK) cells, tumor-associated macrophages (TAMs), neutrophils, dendritic cells, and myeloid-derived suppressor cells (MDSCs) (132, 138). The proportions and functional states of these cell populations vary substantially between tumors and are influenced by the composition of the gastric microbiota (134, 139, 140).

CD8+ T-cell exhaustion is a hallmark of the immunosuppressive gastric TIME. Exhausted CD8+ T cells are characterized by high expression of inhibitory receptors including PD-1, TIM-3, LAG-3, and CTLA-4, and by reduced production of effector cytokines such as IFN-γ, TNF-α, and granzyme B (141, 142). The gastric microbiota has been implicated in driving CD8+ T-cell exhaustion via several mechanisms. *H. pylori* infection upregulates PD-L1 expression on gastric epithelial cells via the activation of NF-κB and STAT3 signaling pathways, creating a PD-L1-rich microenvironment that promotes T-cell exhaustion (143, 144) Beyond *H. pylori*, gastric microbiome alterations in GC patients, including the enrichment of F. nucleatum, have been associated with increased infiltration of exhausted CD8^+^ T cells and reduced CD8^+^ tissue-resident memory T cells—a population critical for antitumor immunosurveillance—suggesting that microbial dysbiosis may directly impair immune control of tumor growth (145, 146).

Macrophage polarization represents another key axis of gastric microbiota-driven immune remodeling. TAMs in GC predominantly exhibit an M2-like phenotype characterized by high expression of CD163, CD206, and IL-10, and low expression of pro-inflammatory cytokines and antigen-presenting molecules (145, 147, 148). M2-polarized macrophages promote tumor progression through the secretion of immunosuppressive cytokines (IL-10, TGF-β), angiogenic factors (VEGF), and matrix metalloproteinases that facilitate tumor invasion and metastasis (130, 149, 150). The gastric microbiota influences macrophage polarization through multiple mechanisms, including TLR-mediated sensing of microbial ligands and the effects of microbial metabolites. *H. pylori* infection promotes M2 macrophage polarization through the activation of STAT3 and the induction of arginase-1 expression (56, 103). Lactic acid, produced by Lactobacillus species enriched in the GC microbiota, promotes M2 polarization via the MCT-HIF1α signaling axis, linking gastric microbial metabolism to the immunosuppressive TIME (151–153).

MDSCs represent a third major immunosuppressive cell population in the gastric TIME. They constitute a heterogeneous population of immature myeloid cells that expand in the tumor-bearing host and suppress T-cell proliferation and effector function through the production of arginase-1, inducible nitric oxide synthase (iNOS), ROS, and immunosuppressive cytokines (154–156). The expansion of MDSCs in GC has been associated with advanced disease stage, poor prognosis, and reduced efficacy of immunotherapy (148, 157). The gastric microbiota may contribute to MDSC expansion through the chronic stimulation of myelopoiesis by microbial products, including LPS, that engage TLR4 on hematopoietic progenitor cells and promote differentiation of MDSCs at the expense of mature myeloid cells (154, 158, 159). The enrichment of Gram-negative bacteria in the dysbiotic gastric microbiota may therefore sustain MDSC expansion through chronic delivery of TLR4 agonists to the bone marrow and TIME (160).

The regulatory T cell (Treg) compartment is also modulated by the gastric microbiota. Tregs, characterized by CD4+CD25+FoxP3+ expression, suppress effector T-cell responses through the production of IL-10 and TGF-β as well as contact-dependent mechanisms (161, 162). The density of tumor-infiltrating Tregs is increased in GC and is associated with poor prognosis (163, 164). The gastric microbiota influences Treg differentiation through the generation of microbial metabolites, including SCFAs, that promote FoxP3 expression and Treg expansion (121, 122). *H. pylori* promotes differentiation of Tregs through induction of tolerogenic dendritic cells that express IL-10 and TGF-β, contributing to establishment of a tolerogenic gastric immune environment that facilitates persistent infection (56, 103, 108).

### 4.2 Microbial metabolites as immune modulators

Microbial metabolites serve as a critical molecular interface through which the gastric microbiota shapes the TIME. SCFAs, particularly butyrate and propionate, exert potent immunomodulatory effects (121, 122, 126). Butyrate inhibits histone deacetylases (HDACs) in T cells, promoting acetylation of histone H3 at the FoxP3 locus and enhancing FoxP3 expression, thereby driving Treg differentiation (121, 123, 124). Butyrate also modulates dendritic cell maturation by inhibiting expression of co-stimulatory molecules (CD80, CD86) and MHC class II, reducing the capacity of dendritic cells to prime effector T-cell responses (165, 166). In the gastric context, the effects of butyrate are likely to be complex and concentration-dependent. At low concentrations, butyrate may promote a tolerogenic immune environment that facilitates tumor immune evasion. At high concentrations, butyrate can induce apoptosis in colorectal cancer cells and may have context-dependent antitumor effects in the gastric epithelium (121–123, 167).

Secondary bile acids are potent immunomodulatory molecules. Deoxycholic acid (DCA), the most abundant secondary bile acid in the human gastrointestinal tract, activates the farnesoid X receptor (FXR) and the G protein-coupled bile acid receptor TGR5 on immune cells, modulating inflammatory and immune responses (126, 127). DCA promotes Th17 cell differentiation and suppresses Treg differentiation, shifting the Th17/Treg balance toward a pro-inflammatory state that may paradoxically contribute to tumor-promoting inflammation in the gastric microenvironment (83, 126). DCA also induces production of ROS and DNA damage in epithelial cells, contributing to the genotoxic axis of carcinogenesis (119).

Polyamines, including putrescine, spermidine, and spermine, are produced by bacterial decarboxylation of amino acids and are present at elevated concentrations in the TIME (83, 90, 122). They modulate immune cell function through multiple mechanisms, including the inhibition of pro-inflammatory cytokine production by macrophages and the promotion of M2 macrophage polarization (118, 122, 126). The enrichment of polyamine-producing bacteria in the gastric microbiota may therefore contribute to the immunosuppressive TIME via sustained delivery of polyamines to tumor-infiltrating immune cells (128, 129).

### 4.3 Microbiota-immunotherapy interactions in gastric cancer

The impact of the gastric microbiota on the efficacy of immune checkpoint inhibitors (ICIs) represents one of the most clinically significant dimensions of the DCIT framework (135, 136, 168). ICIs targeting the PD-1/PD-L1 and CTLA-4 axes have transformed the treatment landscape for advanced GC, with the anti-PD-1 antibodies nivolumab and pembrolizumab now approved for use in various GC treatment settings (149, 169, 170). However, only a subset of GC patients responds to ICI therapy, and the identification of predictive biomarkers and strategies to convert non-responders to responders remains a major clinical priority (156, 171, 172).

The gut microbiota has been established as a modulator of ICI efficacy in multiple cancer types, including melanoma, non-small cell lung cancer, and renal cell carcinoma (173–175). The evidence for gastric microbiota modulation of ICI efficacy in GC is more limited but is emerging from preclinical models and clinical cohort studies (172, 176, 177). The paradoxical relationship between *H. pylori* seropositivity and ICI response has attracted particular interest. Several retrospective studies have reported that *H. pylori*-seropositive patients with various cancers, including non-small cell lung cancer and melanoma, have reduced response rates to anti-PD-1 therapy compared with seronegative patients (178–180). This observation has been attributed to *H. pylori*-induced systemic immunosuppression, including the expansion of Tregs and the induction of tolerogenic dendritic cells, which may limit the ability of the immune system to mount an effective antitumor response even after PD-1 blockade (84, 161). However, the relationship between *H. pylori* status and ICI efficacy in GC specifically remains poorly characterized, and prospective studies are needed to clarify this association (180–182).

The broader gastric microbiota composition, beyond *H. pylori*, also influences ICI efficacy (168, 183, 184). The enrichment of specific bacterial taxa, including Bifidobacterium and Akkermansia, in the gut microbiota has been associated with improved ICI responses in multiple cancer types, and these taxa may exert similar effects in the gastric microenvironment (180, 185). The mechanisms by which these bacteria enhance ICI efficacy include the production of SCFAs that promote CD8+ T-cell effector function, the activation of STING signaling in dendritic cells, and the cross-presentation of tumor antigens (121, 173). Conversely, the enrichment of immunosuppressive taxa, including certain Bacteroides and Fusobacterium species, has been associated with reduced ICI efficacy, potentially through the promotion of Treg expansion and the suppression of CD8+ T-cell responses (179, 186).

The concept of microbiota-informed immunotherapy has emerged as a promising strategy to enhance the efficacy of ICIs in GC (187, 188). Approaches under investigation include co-administration of probiotics or defined bacterial consortia with ICIs, fecal microbiota transplantation (FMT) from ICI-responding donors to non-responding recipients, and modulation of the gastric microbiota through dietary interventions or antibiotics (185, 189, 190). The clinical translation of these approaches is at an early stage, and several challenges must be addressed, including the identification of the optimal bacterial strains or consortia for combination with ICIs, the standardization of microbiota-modulating interventions, and the assessment of safety in immunocompromised patients (191, 192).

The challenges and opportunities in microbiota-informed immunotherapy for GC, outlined in **Table 2**, reflect the broader translational hurdles that must be overcome to realize the clinical potential of the DCIT framework (168). The *H. pylori* paradox, in which *H. pylori* seropositivity correlates with reduced ICI response in some studies, requires prospective validation in GC-specific cohorts (176, 177). Considerable inter-individual variability in gastric microbiota composition, even among patients with similar disease stages, complicates the identification of universal microbial signatures predictive of ICI response. Establishing causality, rather than correlation, between microbiota composition and ICI efficacy requires the development of experimental models that faithfully recapitulate the human gastric TIME (191, 193–195).

**Table 2:** Challenges in microbiota-informed immunotherapy for gastric cancer.

Challenge	Manifestation	Current strategies	Future directions
*H. pylori* paradox	Seropositivity linked to reduced ICI response	Retrospective cohort analysis	Prospective trials with *H. pylori* stratification
Microbial heterogeneity	Inter-individual variability in microbiota composition	Fecal/stool metagenomic profiling	Standardized gastric sampling protocols
Causal inference	Correlative microbiota-ICI response associations	Gnotobiotic mouse models	Human organoid-microbiota co-culture systems
Safety in immunocompromised hosts	Risk of bacteremia with live biotherapeutics	Probiotic safety screening	Engineered auxotrophic strains
Biomarker validation	No validated microbiota-based predictive biomarkers	Exploratory multi-omics analyses	Prospective biomarker-driven clinical trials

The three-layer immune remodeling model integrates the cellular, metabolic, and checkpoint-modulatory dimensions of gastric microbiota-driven immune reprogramming (**Figure 3**) (114, 116, 130). The inner layer reflects the direct effects of microbial communities on the composition and functional polarization of tumor-infiltrating immune cells, including CD8+ T-cell exhaustion, M2 macrophage polarization, and MDSC expansion (115, 147, 154). The middle layer comprises the molecular mediators of immune remodeling, including microbial metabolites that act as interkingdom signaling molecules capable of rewiring T-cell differentiation and antigen-presenting cell function. The outer layer represents the clinical interface between gastric microbiota composition and checkpoint inhibitor efficacy, where the translation of microbiota insights into actionable therapeutic strategies is most direct. The arrows connecting these layers underscore the bidirectional nature of microbiota-immune interactions: the microbial community shapes the immune landscape, and the immune landscape, which in turn selects for microbial taxa capable of surviving and thriving in the immunosuppressive TIME.

**Figure 3:**
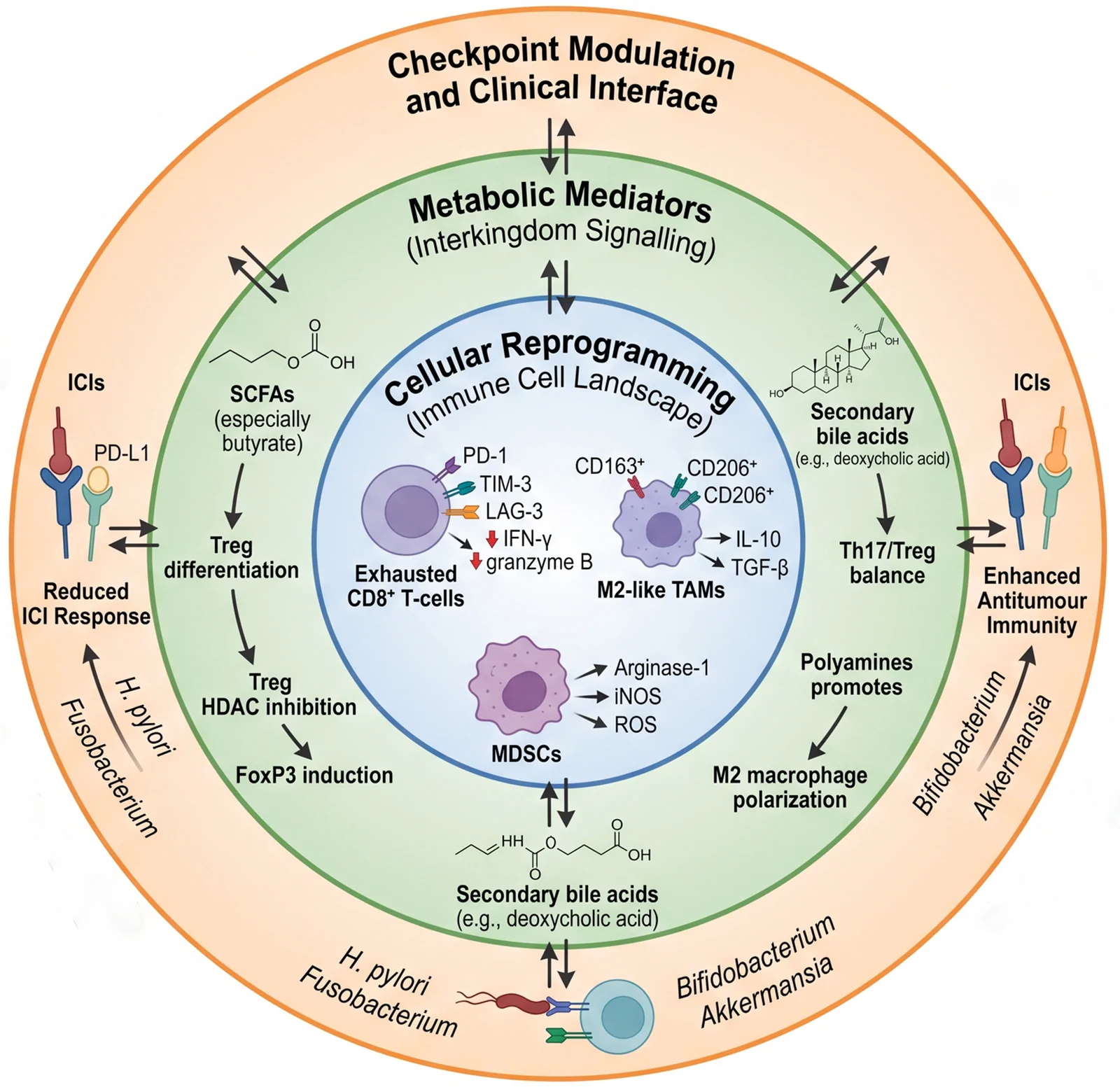
Three-layer model of gastric microbiota-driven immune remodeling. The inner layer represents cellular reprogramming (CD8^+^ T-cell exhaustion, M2 macrophage polarization, MDSC and Treg expansion). The middle layer shows metabolite-mediated signaling (SCFAs, secondary bile acids, polyamines). The outer layer reflects checkpoint modulation and its impact on immunotherapy efficacy. Arrows denote bidirectional interactions between layers and feedback to the microbial community.

## 5 Therapeutic opportunities targeting the gastric microbiota

The fourth dimension of the DCIT framework addresses translation of microbiota insights into therapeutic strategies for gastric cancer prevention and treatment. The therapeutic pipeline spans a continuum from established interventions with proven clinical benefit to emerging modalities at the preclinical or early clinical stage. This section evaluates the evidence for each therapeutic modality, emphasizing the contrast between preclinical promise and clinical validation (**Figure 4**).

**Figure 4:**
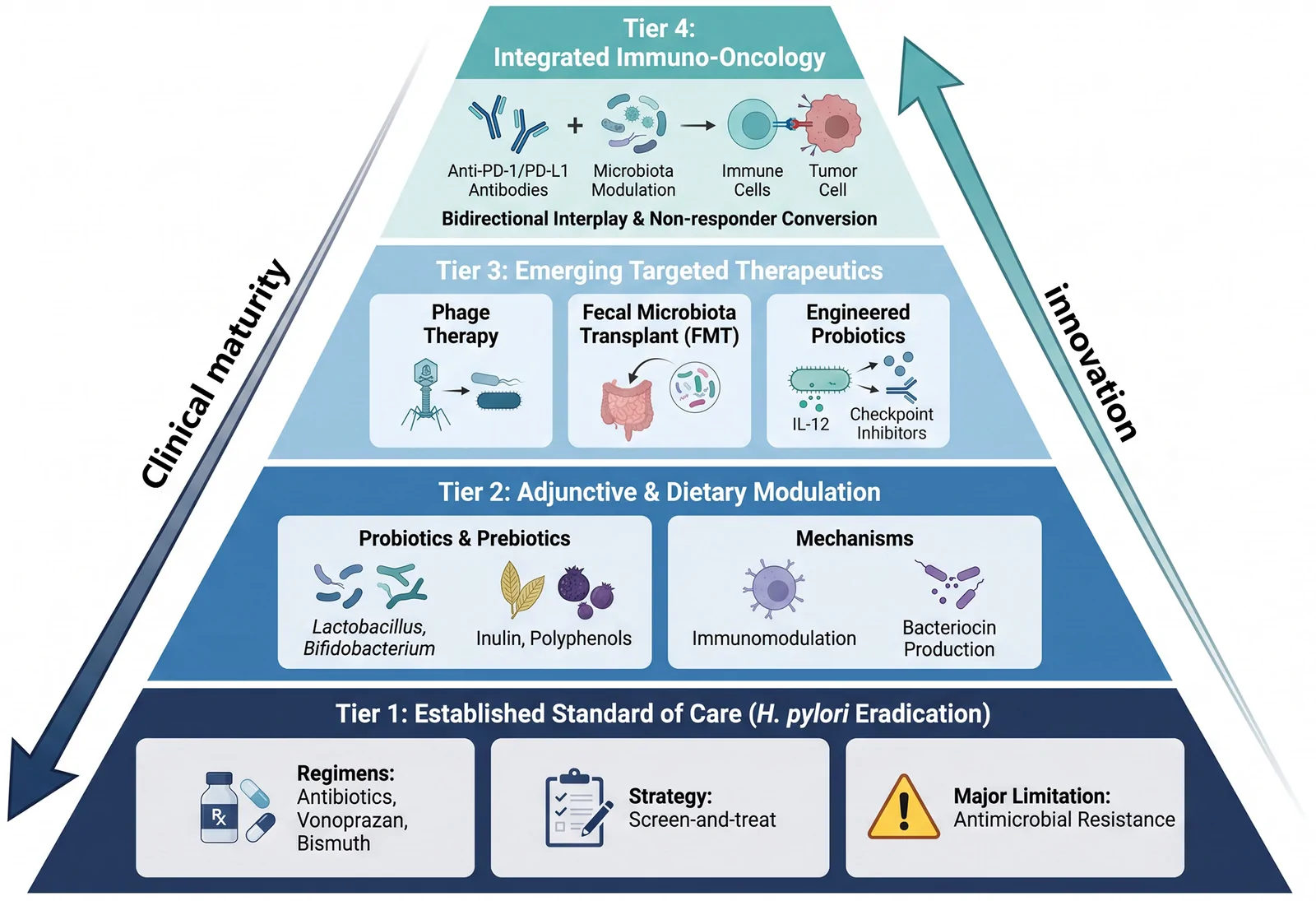
Therapeutic pipeline targeting the gastric microbiota in gastric cancer. This figure depicts a hierarchical framework of microbiota-directed interventions organized by clinical maturity and mechanistic specificity within the DCIT framework. Tier 1 (standard of care) covers *H. pylori* eradication regimens (vonoprazan-based, bismuth quadruple, clarithromycin-containing), with major constraints including antimicrobial resistance and incomplete risk elimination. Tier 2 (adjunctive) includes probiotics, prebiotics, and dietary polyphenols that improve eradication and reduce side effects but lack direct cancer-prevention evidence. Tier 3 (emerging) comprises phage therapy, fecal microbiota transplantation, and engineered probiotics for selective bacterial depletion or targeted payload delivery, all at preclinical/early clinical stages. Tier 4 (integration) highlights microbiota-informed strategies combined with immune checkpoint inhibitors, exploiting bidirectional microbiota-immunity crosstalk. Color gradients indicate decreasing clinical maturity (green to orange) and increasing innovation. Key translational gaps such as causality validation, biomarker stratification, and standardized protocols are annotated as prerequisites for clinical implementation.

### 5.1 *H. pylori* eradication in the era of antimicrobial resistance

*H. pylori* eradication remains the cornerstone of gastric cancer prevention, supported by randomized controlled trials (RCTs) demonstrating that eradication reduces the incidence of metachronous GC after endoscopic resection of early gastric cancer and, in population-based studies, reduces the risk of GC development in asymptomatic individuals (10, 11, 196, 197). The standard-of-care eradication regimen has evolved from clarithromycin-based triple therapy (PPI, clarithromycin, amoxicillin or metronidazole) to bismuth quadruple therapy (PPI, bismuth, tetracycline, metronidazole) and, more recently, to vonoprazan-based regimens, driven by the global increase in clarithromycin resistance (198–200).

The rise of antimicrobial resistance poses the most significant threat to the effectiveness of *H. pylori* eradication as a GC prevention strategy. In many regions of Asia, Europe, and the Americas, clarithromycin resistance rates have exceeded 15%, the threshold above which clarithromycin-based triple therapy is no longer recommended (198, 201, 202). Metronidazole resistance is even more widespread, with rates exceeding 40% in some populations, although the clinical impact of in vitro metronidazole resistance is partially mitigated by the acidic gastric environment, which enhances metronidazole activity (197, 199, 200, 203). Levofloxacin resistance has also increased, limiting the utility of levofloxacin-based rescue regimens (204, 205). The emergence of multidrug-resistant *H. pylori* strains highlights the need for antimicrobial stewardship and the development of non-antibiotic-based eradication strategies (198, 206).

Vonoprazan, a potassium-competitive acid blocker that provides more potent and sustained acid suppression than PPIs, has become an important component of modern *H. pylori* eradication regimens (207–209). Vonoprazan-based triple therapy (vonoprazan, amoxicillin, clarithromycin) shows superior eradication rates compared with PPI-based triple therapy in regions with high clarithromycin resistance, and vonoprazan-based dual therapy (vonoprazan, amoxicillin) has shown promise as a simplified regimen with reduced antibiotic exposure (210–212). The integration of vonoprazan into standard eradication protocols is likely to improve eradication rates and reduce the selective pressure for antimicrobial resistance (213, 214).

The impact of *H. pylori* eradication on the non-*H. pylori* gastric microbiota is an important consideration. Eradication therapy, particularly when containing broad-spectrum antibiotics, can transiently disrupt the gastric and intestinal microbiota, reducing microbial diversity and altering the relative abundance of specific taxa (215, 216). The long-term consequences of eradication-induced microbiota alterations are not fully understood, but studies indicate that the gastric microbiota of successfully eradicated individuals partially recovers toward a *H. pylori*-negative composition over time, albeit with persistent differences compared with never-infected individuals (47, 217). The selection of antibiotic-resistant strains in the gut microbiota during eradication therapy is an additional concern, particularly given the high rates of antibiotic resistance in many populations (198, 200).

The “screen-and-treat” strategy, in which asymptomatic individuals are screened for *H. pylori* infection and treated if positive, has been evaluated in population-based studies, most notably in Japan, where a national *H. pylori* eradication program was introduced in 2013 (10, 11, 196). Although the program has been associated with a reduction in *H. pylori* prevalence and, in some analyses, a decline in GC mortality, the overall impact on GC incidence has been less dramatic than anticipated, reflecting the long latency period between *H. pylori* infection and GC development and the fact that many individuals in the screened population had already developed preneoplastic lesions at the time of eradication (196, 218). These findings highlight the importance of early eradication, ideally before the development of atrophic gastritis, to maximize the GC-preventive benefit (219).

### 5.2 Probiotics, prebiotics, and dietary microbiota modulation

Probiotic supplementation represents the most accessible microbiota-modulating intervention for gastric cancer prevention and has been studied primarily in the context of *H. pylori* eradication and gastritis management (219–222). Lactobacillus, Bifidobacterium, and the yeast Saccharomyces boulardii are the most extensively studied probiotic organisms in the gastric context (223–226). Meta-analyses of RCTs show that probiotic supplementation during *H. pylori* eradication therapy modestly improves eradication rates (by approximately 5-10%) and significantly reduces the incidence of antibiotic-associated gastrointestinal side effects, including diarrhea, bloating, and nausea (219, 227, 228). The mechanisms underlying these benefits include direct inhibition of *H. pylori* growth by probiotic-derived bacteriocins and organic acids, competition for epithelial adhesion sites, and immunomodulation that reduces gastric inflammation (220, 222, 229–232).

The evidence for probiotic-mediated direct prevention of gastric carcinogenesis is less robust. In animal models, administration of Lactobacillus and Bifidobacterium strains reduces the incidence of *H. pylori*-induced gastric preneoplastic lesions and attenuates gastric inflammation (220, 222, 233). However, RCTs in humans have not yet demonstrated a reduction in GC incidence with probiotic supplementation, and the existing evidence is limited to intermediate endpoints such as the regression of atrophic gastritis and intestinal metaplasia. The variability in probiotic strain selection, dosing, and duration of administration across studies complicates the interpretation of the available evidence (222, 223, 225).

Prebiotics, including inulin, fructo-oligosaccharides (FOS), and galacto-oligosaccharides (GOS), are non-digestible dietary fibers that selectively stimulate the growth of beneficial gut bacteria, particularly Bifidobacterium and Lactobacillus species. The rationale for prebiotic use in gastric cancer prevention is based on the premise that prebiotic-driven expansion of SCFA-producing bacteria in the gut increases the delivery of butyrate and other immunomodulatory SCFAs to the gastric mucosa, thereby reducing inflammation and potentially attenuating carcinogenesis (166, 234). However, clinical evidence for the efficacy of prebiotics in gastric cancer prevention is lacking, and the effects of prebiotics on the gastric microbiota specifically, as opposed to the colonic microbiota, have not been well characterized.

Dietary polyphenols, including flavonoids, phenolic acids, and stilbenes, are plant-derived compounds with anti-inflammatory, antioxidant, and antimicrobial properties that have been studied for their potential to modulate the gastric microbiota and reduce GC risk (235–237). Polyphenols have been shown to inhibit *H. pylori* growth in vitro and to reduce *H. pylori*-induced gastric inflammation in animal models (237–240). Epidemiological studies report inverse associations between dietary polyphenol intake and GC risk, although the evidence is primarily from case-control studies and subject to confounding by other dietary and lifestyle factors (241–243). The mechanisms by which polyphenols modulate the gastric microbiota include direct antimicrobial activity against *H. pylori* and other gastric pathogens, prebiotic-like stimulation of beneficial bacteria, and the modulation of bacterial quorum sensing and virulence gene expression (244–246). The clinical translation of dietary polyphenol interventions for GC prevention is limited by the low bioavailability of many polyphenols and the variability in polyphenol metabolism by the gut microbiota.

### 5.3 Phage therapy, FMT, and engineered microbial therapeutics

The limitations of conventional antibiotic-based *H. pylori* eradication and the challenges of achieving sustained gastric microbiota restoration with probiotics have driven the development of more targeted and sophisticated microbiota-modulating interventions (247, 248). Bacteriophage therapy, FMT, and engineered probiotic strains represent the frontier of gastric microbiota therapeutics, offering unprecedented selectivity and programmability (189, 191, 249, 250).

Bacteriophages are viruses that infect and lyse specific bacterial hosts, and phage therapy has been proposed as a strategy for the selective depletion of carcinogenic bacteria from the gastric microbiota without the collateral damage to commensal communities associated with broad-spectrum antibiotics (247, 251, 252). The advantages of phage therapy include high host specificity, self-amplification at the site of infection, and a favorable safety profile (253). Phages targeting *H. pylori* have been isolated and characterized, and preclinical studies indicate that phage administration can reduce *H. pylori* colonization in mouse models (254–256). However, the clinical translation of phage therapy for gastric applications faces several challenges, including the need for acid-stable phage formulations that can survive gastric transit, the potential for phage resistance to develop in the target bacterial population, and the regulatory hurdles associated with the approval of phage-based therapeutics (257, 258).

FMT, which involves the transfer of fecal microbiota from a healthy donor to a recipient, has been established as a highly effective treatment for recurrent Clostridioides difficile infection and is being investigated for a range of other conditions, including inflammatory bowel disease and metabolic syndrome (185, 187). The rationale for FMT in the context of gastric cancer is based on the hypothesis that the transplantation of a healthy gastric microbiota, or a healthy gut microbiota that indirectly influences the gastric ecosystem, could restore microbial homeostasis and reduce carcinogenic risk (188, 259, 260). The landmark study by Kwon and colleagues demonstrated that human gastric microbiota transplantation from patients with intestinal metaplasia recapitulated premalignant lesions in germ-free mice, providing direct evidence that the gastric microbiota can drive development of preneoplastic pathology (193). This study also suggests the corollary that transplantation of a healthy gastric microbiota could reverse preneoplastic changes, although this hypothesis has not been tested in clinical trials (260, 261).

Engineered probiotics represent a rapidly advancing field in which probiotic strains are genetically modified to deliver therapeutic payloads, including antitumor cytokines, checkpoint inhibitors, or tumor antigens, directly to the TIME (189, 191). Lactobacillus and Bifidobacterium strains have been engineered to express IL-2, IL-12, and other immunostimulatory cytokines, and preclinical studies demonstrate that these engineered strains can enhance antitumor immunity and reduce tumor growth in mouse models of gastric and colorectal cancer (189, 190, 257). The advantages of engineered probiotics include specific targeting of the TIME, the sustained local production of therapeutic molecules, and the potential for oral administration (191, 192, 262). The safety concerns associated with the administration of live, genetically modified bacteria to immunocompromised cancer patients, including the risk of bacteremia and the potential for horizontal gene transfer of antibiotic resistance genes, must be rigorously addressed before clinical translation.

### 5.4 Integrating microbiota modulation with standard cancer therapy

The integration of microbiota modulation with conventional chemotherapy, radiotherapy, and immunotherapy represents a pragmatic approach to leveraging the therapeutic potential of the gastric microbiota within existing treatment paradigms (160, 165, 166). The rationale for this integration is based on two complementary observations: first, that chemotherapy and radiotherapy induce gastrointestinal dysbiosis that can exacerbate treatment-related toxicity and potentially compromise treatment efficacy; and second, that the composition of the gut and gastric microbiota can influence the efficacy of chemotherapy and immunotherapy via modulation of drug metabolism, immune responses, and the TIME (135, 160, 165).

Chemotherapy-induced dysbiosis is a well-characterized phenomenon in which the administration of cytotoxic agents, particularly 5-fluorouracil and platinum-based drugs, reduces the diversity of the gut microbiota and enriches potentially pathogenic taxa (135, 160, 165, 263). This dysbiosis contributes to the development of chemotherapy-induced mucositis, diarrhea, and systemic inflammation, which can limit treatment tolerability and necessitate dose reductions or treatment delays (264). Probiotic supplementation during chemotherapy reduces the incidence and severity of treatment-related diarrhea in several RCTs, although the evidence is not yet sufficient to support routine clinical recommendation (166, 223–226).

As discussed in the preceding section on immune remodeling, the gastric microbiota can modulate immunotherapy efficacy. The integration of microbiota modulation with ICI therapy is particularly promising, given the evidence that the microbiota composition can predict and modulate ICI efficacy (265–267). Clinical trials are currently evaluating the co-administration of defined bacterial consortia or FMT with anti-PD-1 therapy in patients with advanced GC and other solid tumors (180, 185, 187, 188, 260). The results of these trials will inform the clinical feasibility and efficacy of microbiota-informed immunotherapy and may establish the foundation for a new class of adjunctive cancer therapies.

The therapeutic modalities summarized in **Table 3** span a wide range of clinical maturity, from established interventions with proven efficacy to emerging modalities at the preclinical stage (2, 21–23). The contrast between the strength of preclinical evidence and the paucity of RCT data for most non-eradication modalities characterizes the current therapeutic landscape (135, 136). Addressing this evidence gap will require well-designed clinical trials that incorporate biomarker-guided patient stratification, standardized microbiota-modulating interventions, and clinically meaningful endpoints.

**Table 3:** Therapeutic modalities targeting the gastric microbiota in gastric cancer.

Modality	Mechanism	Clinical stage	Key evidence	Limitations
*H. pylori* eradication	Antibiotic clearance of carcinogenic bacteria	Standard of care	RCTs showing reduced GC risk (10, 11, 196)	Antimicrobial resistance (198, 268)
Probiotics	Microbiota restoration, immunomodulation	Phase II/III	Improved eradication rates, reduced side effects (219, 227)	Strain selection, limited GC prevention data (220, 222)
Phage therapy	Selective bacterial depletion	Preclinical	*H. pylori* reduction in mouse models (247)	Acid stability, phage resistance (247, 269)
FMT	Gastric ecosystem restoration	Preclinical/early clinical	Premalignant lesion recapitulation in mice (193)	Safety, donor screening, standardization (259, 270)
Engineered probiotics	Targeted delivery of antitumor payloads	Preclinical	Tumor reduction in mouse models (23, 190)	Safety in immunocompromised hosts (189, 191)
Dietary polyphenols	Antimicrobial, anti-inflammatory	Observational	Reduced GC risk in epidemiological studies (123, 237)	Low bioavailability, confounding (123, 236)

## 6 Multi-omics integration and clinical translation: gaps and evidence-strength assessment

The translation of gastric microbiota research into clinical practice requires not only mechanistic understanding but also robust methodological frameworks for data generation, integration, and interpretation (17, 20, 72, 271). This section critically evaluates the methodological landscape and identifies the unresolved research gaps that need to be addressed to realize the translational potential of the DCIT framework.

The integration of multi-omics platforms has become a powerful approach for comprehensive characterization of the gastric microbial ecosystem and its host interactions (272–274). Metagenomics provides taxonomic and functional gene content information at species-level resolution, enabling the identification of microbial pathways that are enriched or depleted in GC (73–75). Metatranscriptomics captures the actively expressed gene repertoire of the gastric microbiota, thereby distinguishing metabolically active community members from dormant or transient taxa (17, 72). Metabolomics, using targeted and untargeted approaches, profiles the small-molecule metabolites produced by the combined activity of the gastric microbiota and the host, offering a functional readout of microbial metabolism that is directly relevant to the carcinogenic mechanisms discussed earlier (275–277).

The integration of these omics layers with single-cell and spatial transcriptomics has begun to reveal the spatial organization of the gastric TIME and its relationship to the microbial communities previously identified as universal signatures of dysbiosis in gastric carcinogenesis (278–283). Recent spatially resolved transcriptomic atlases of gastric cancer have provided unprecedented insights into the architectural organization of the TIME, including the identification of lymphocyte-aggregated regions enriched for B cells and T cells, which correlate with distinct clinical outcomes and immune checkpoint expression patterns (131, 133, 134, 284). Similarly, Sun et al. (2023) combined spatial transcriptomics with metabolomics to reveal cell-specific metabolic reprogramming and intercellular communication networks that vary across tumor subregions (285). These studies underscore that the immune landscape is not uniform; rather, it exhibits pronounced spatial heterogeneity that may be influenced by local microbial colonization. Although direct spatial profiling of gastric microorganisms within the tumor tissue remains technically challenging, emerging evidence suggests that bacterial biogeography, particularly the differential distribution of mucosa-adherent versus luminal bacteria, can shape regional immune cell polarization, such as M2-like macrophage accumulation in areas of high F. nucleatum abundance (32). Integrating microbial spatial mapping with single-cell and spatial transcriptomics will be essential to dissect how the physical proximity of specific bacteria to immune cells dictates local immunosuppression or activation. Such integrative approaches are now beginning to be applied in gastrointestinal cancers (282, 286) and hold promise for identifying spatially defined biomarkers that predict immunotherapy response in gastric cancer.

The low-biomass nature of gastric samples remains a fundamental methodological challenge that demands rigorous contamination control. The microbial biomass of gastric biopsies is typically several orders of magnitude lower than that of fecal samples, making gastric microbiome studies particularly susceptible to contamination from DNA extraction reagents, PCR reagents, and laboratory consumables. The inclusion of negative controls at every stage of sample processing, from endoscopy to sequencing, is essential to distinguish genuine gastric microbial signals from contaminating sequences. The lack of standardized protocols for gastric microbiome studies, including variability in sampling site (antrum versus corpus), biopsy storage and processing, DNA extraction method, and sequencing platform, complicates cross-study comparisons and limits the generalizability of individual findings.

Establishing causality from correlative microbiome data represents a second major challenge. The majority of gastric microbiota studies to date are cross-sectional, comparing the microbiota composition of GC patients with that of non-cancer controls (264, 287–290). Although these studies have identified consistent microbial signatures associated with GC, they cannot distinguish between microbial alterations that contribute to carcinogenesis and those that are consequences of the altered gastric environment in the presence of cancer. Prospective longitudinal cohort studies, in which the gastric microbiota is characterized before the development of GC, are needed to establish the temporal relationship between dysbiosis and carcinogenesis. The use of gnotobiotic animal models, in which the gastric microbiota is experimentally manipulated, has provided causal evidence for the role of specific microbial taxa in gastric carcinogenesis, including the demonstration that human gastric microbiota transplantation recapitulates premalignant lesions in germ-free mice (193–195, 283, 291). However, the translation of findings from animal models to human disease requires caution, given the substantial differences in gastric physiology and immune system function between mice and humans.

Several major knowledge gaps in gastric microbiota research remain. The functional contribution of the gastric mycobiome and virome to carcinogenesis is largely unexplored. The gastric fungal community, dominated by Candida, Saccharomyces, and Aspergillus species, has been shown to be altered in GC, with enrichment of C. albicans reported in some studies (290, 292–299). However, the functional significance of these fungal shifts, particularly their interactions with bacterial communities and their direct contribution to carcinogenesis, remains largely unexplored. Similarly, the gastric virome, encompassing both bacteriophages and eukaryotic viruses beyond Epstein-Barr virus, has received minimal attention. Bacteriophages can shape bacterial community structure and virulence gene transfer, while other viruses may modulate host immune responses, yet their roles in gastric cancer development are virtually unknown (300–303). Addressing these knowledge gaps will require dedicated metagenomic and metatranscriptomic studies that include fungal and viral marker genes, as well as functional experiments in gnotobiotic models.

## 7 Conclusion and future perspectives

The DCIT framework proposed in this review synthesizes the rapidly growing body of evidence base on gastric microbiota-driven carcinogenesis into a coherent, multi-stage, multi-mechanism model that offers therapeutic intervention at multiple nodes. The framework reveals that gastric microbial dysbiosis is not merely a consequence of the changing gastric environment during carcinogenesis but an active driver that amplifies each subsequent stage of the disease process. The compositional shifts along the Correa cascade, from *H. pylori* dominance through the ecological succession of oral commensals and nitrate-reducing taxa, represent the first dimension of the framework and provide the foundation for the carcinogenic and immunomodulatory consequences that follow.

The four complementary mechanistic axes of carcinogenesis, including genotoxicity, inflammation, barrier disruption, and metabolic reprogramming, are progressively activated along the cascade and intersect to form a self-amplifying carcinogenic network. Immune remodeling of the gastric TIME, driven by microbial metabolites, immune cell reprogramming, and checkpoint modulation, constitutes a critical interface between the gastric microbiota and the host antitumor immune response, with direct implications for the efficacy of immunotherapy. The therapeutic pipeline, from established *H. pylori* eradication through probiotics, phage therapy, FMT, and engineered probiotics to integrated microbiota-immunotherapy strategies, provides multiple opportunities for clinical intervention, but translating preclinical promise into clinical benefit remains hampered by substantial evidence gaps.

Several critical evidence gaps remain to be resolved to realize the clinical potential of the DCIT framework. Large-scale, multi-ethnic prospective cohort studies with standardized gastric sampling protocols are needed to establish the temporal relationship between gastric microbial dysbiosis and GC development and to identify microbial biomarkers capable of stratifying individuals by GC risk. Causal inference frameworks that integrate multi-omics data with functional validation in organoid and animal models are essential to move beyond correlative associations and establish the mechanistic basis for microbiota-targeted interventions. Biomarker-driven clinical trials testing microbiota-modulating interventions as adjuncts to immunotherapy represent a particularly promising translational direction, given the emerging evidence that the gastric microbiota influences ICI efficacy. Exploration of the gastric mycobiome and virome as understudied contributors to carcinogenesis may reveal additional therapeutic targets and biomarkers.

Gastric microbiota research is now poised for clinical translation; success depends on interdisciplinary collaboration spanning microbiology, immunology, oncology, bioinformatics, and clinical trial design. The DCIT framework provides a conceptual scaffold for this interdisciplinary effort, organizing the diverse strands of evidence into a unified model that can guide the design of future studies and the development of microbiota-targeted therapies. The ultimate goal is to harness the gastric microbiota as a therapeutic target in the prevention and treatment of gastric cancer, moving from characterization of dysbiosis to the clinical implementation of microbiota-informed precision oncology.
